# Supplementary figures and images for: A simple method of hiPSCs differentiation into insulin-producing cells is improved with vitamin C and RepSox
Source: PLoS One. 2021 Jul 12;16(7):e0254373. doi: 10.1371/journal.pone.0254373 (PMC8274930; doi:10.1371/journal.pone.0254373)

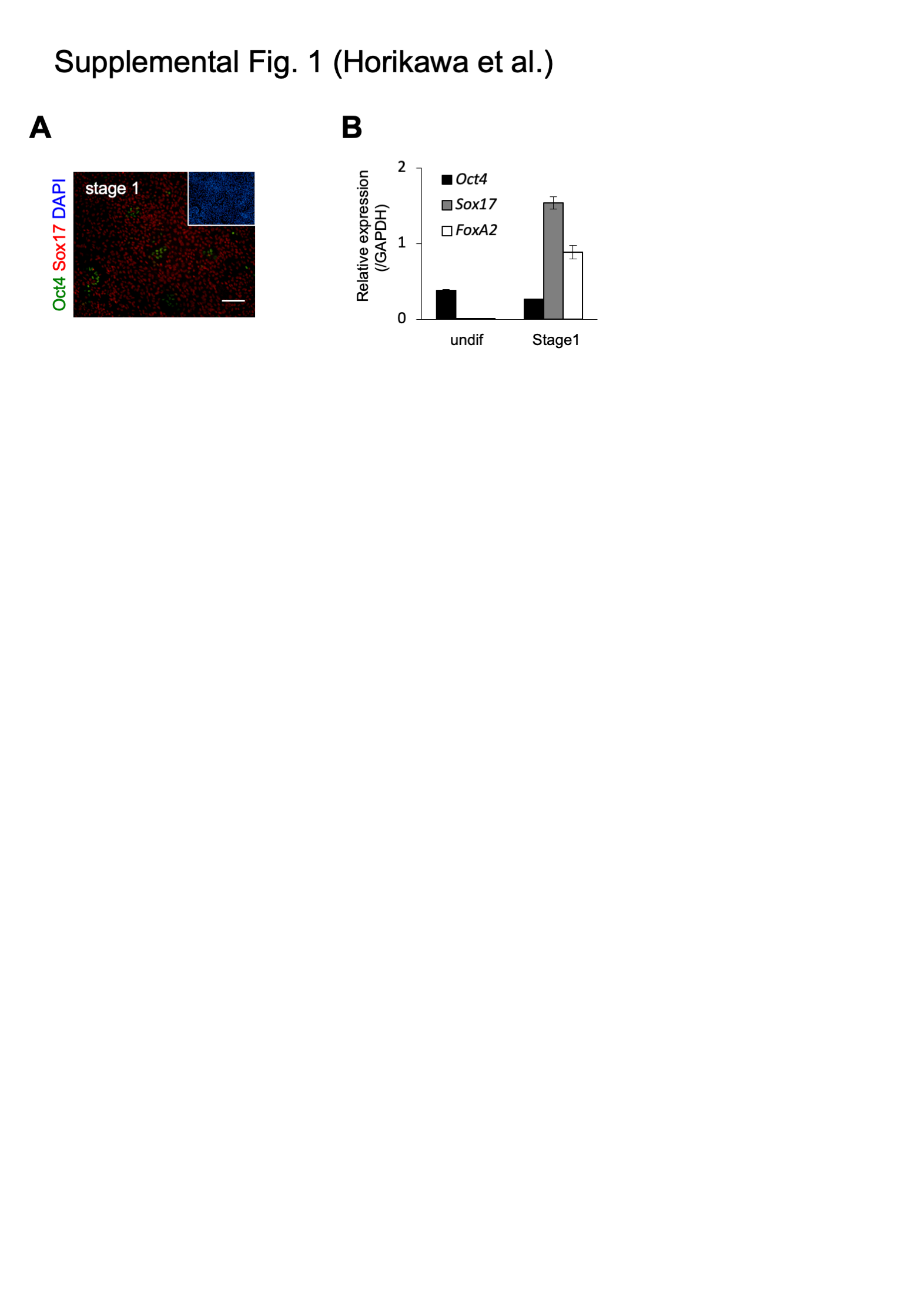

Supplement: S1 Fig — (A) Immunohistochemistry of the cells at stage 1. The expression of Oct4 (green) and Sox17 (red). DAPI staining shown in blue. Scale bar: 100 μm. (B) qRT-PCR analysis of the expression of Oct4 (black), Sox17 (gray), and FoxA2 (white), normalized against GAPDH at stage 1 (three technical replicates per group). (TIF) [file pone.0254373.s001.tif]

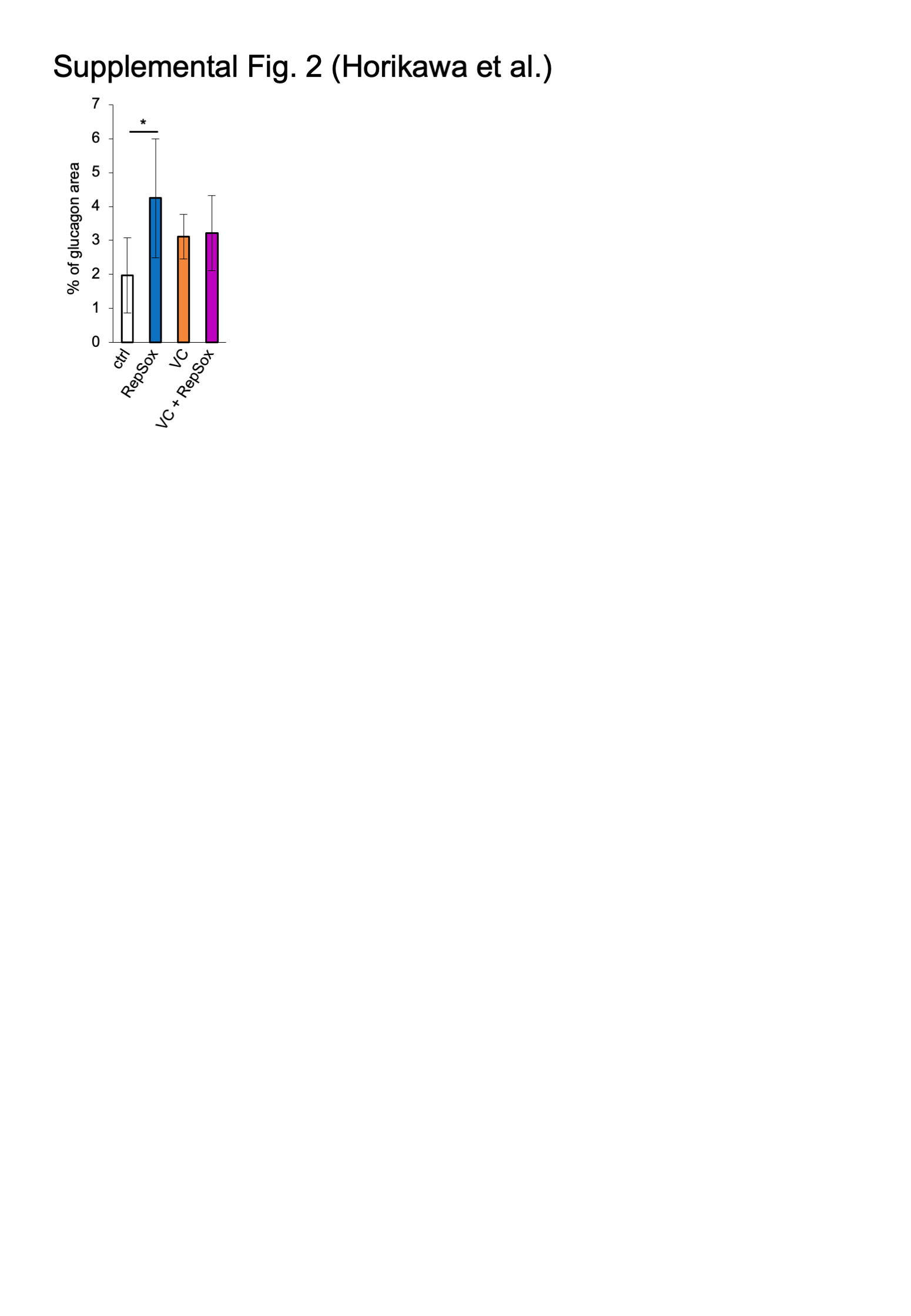

Supplement: S2 Fig — The area corresponds to Fig 4B (n = 5, including one biological and five technical replicates). control, white; RepSox, blue; VC (vitamin C-treated), orange; VC (vitamin C-treated) + RepSox, magenta. *p<0.05, **p<0.01; Welch’s paired t-test. (TIF) [file pone.0254373.s002.tif]

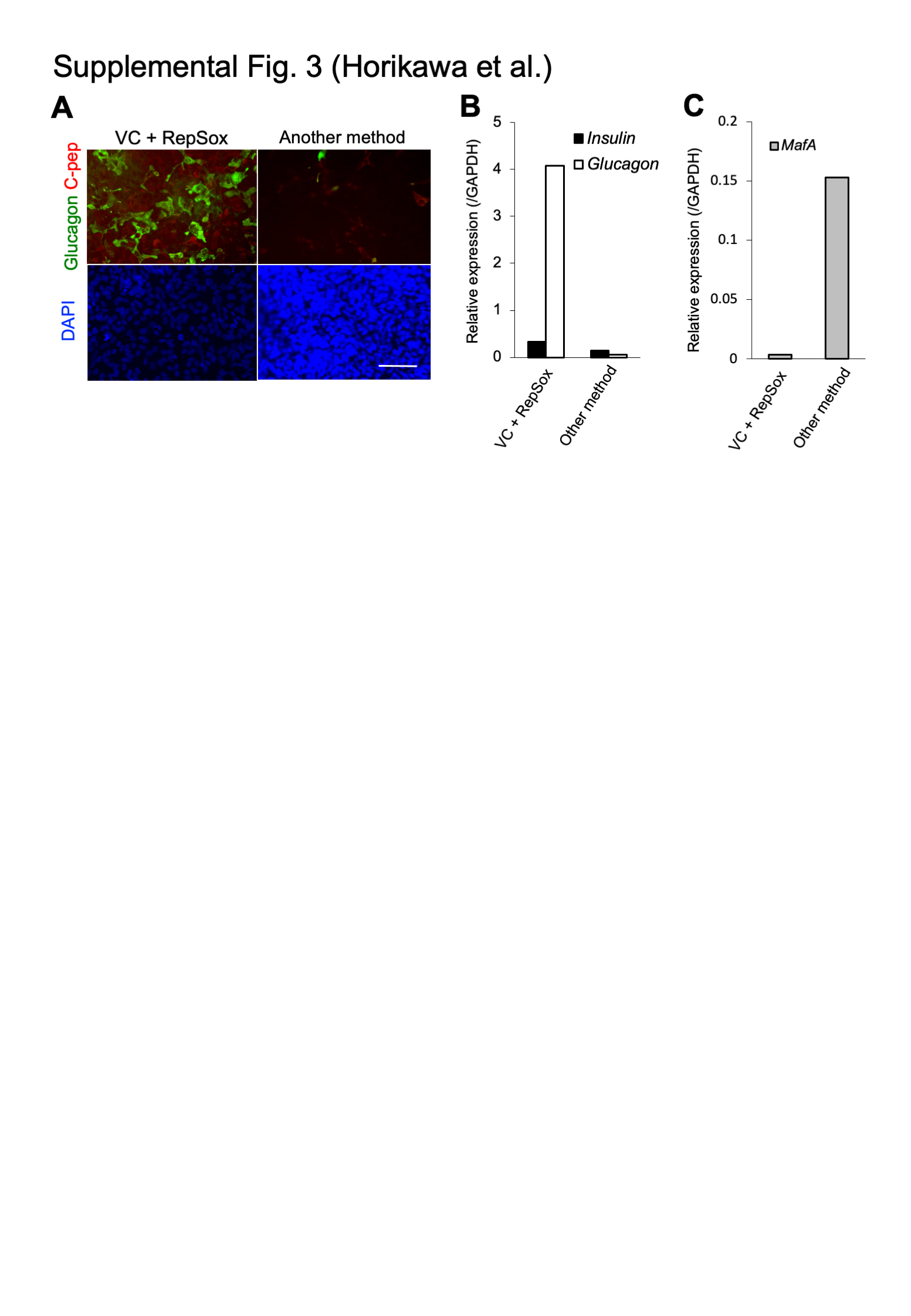

Supplement: S3 Fig — (A) Immunohistochemistory of the cells. The expression of glucagon (green) and C-peptide (red). DAPI staining shown in blue. Scale bar: 100 μm. (B) qRT-PCR analysis of the expression of Insulin (black) and Glucagon (white). (C) qRT-PCR analysis of the expression of MafA (gray). (TIF) [file pone.0254373.s003.tif]

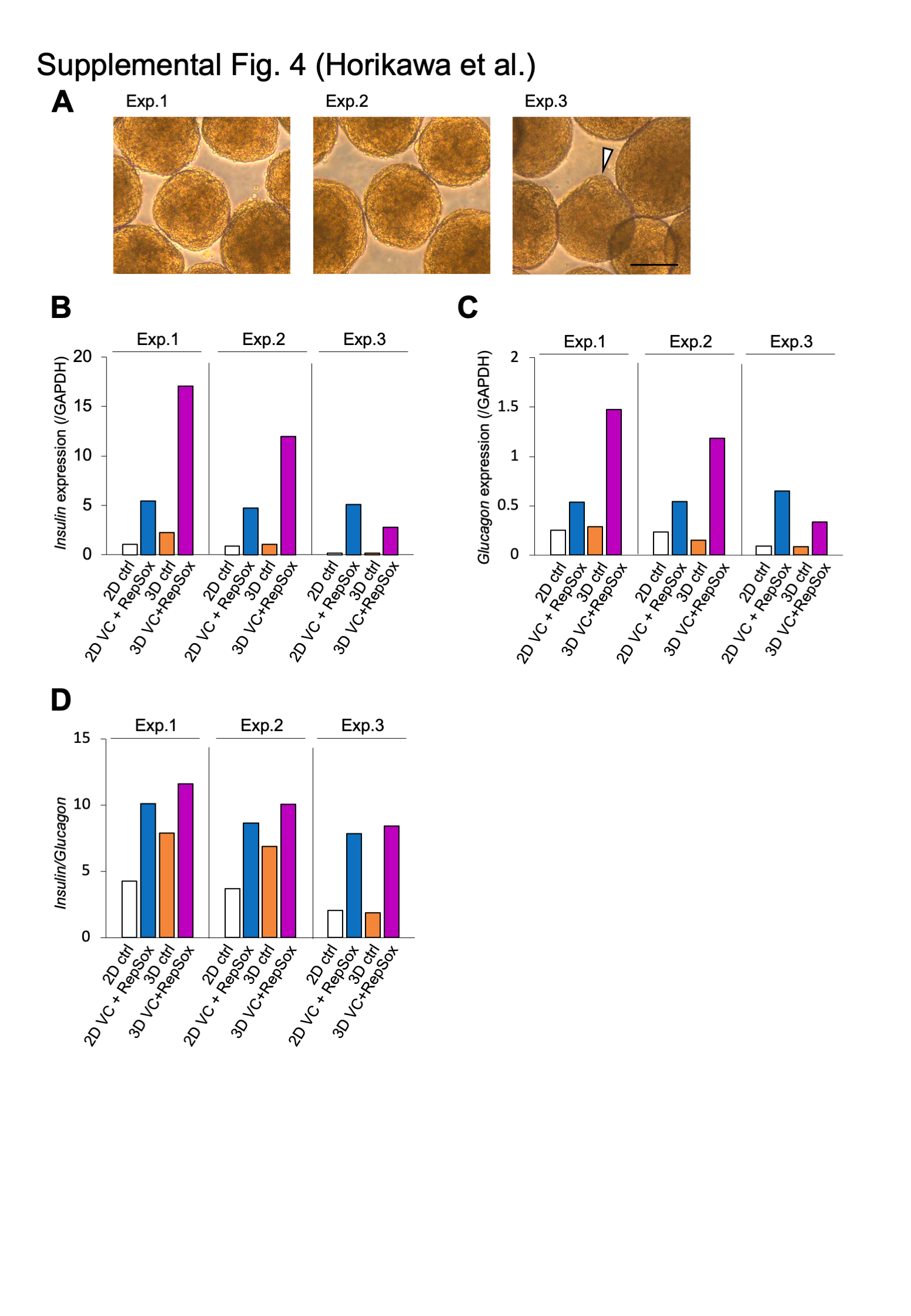

Supplement: S4 Fig — (A) The morphology of spheroids in three independent experiments. The arrowhead indicates a hollow cyst. Scale bar: 100 μm. (B, C, D) qRT-PCR analysis of the expression of insulin (B), glucagon (C) and insulin/glucagon (D), normalized against GAPDH at stage 4, corresponds to Fig 5A. 2D control, white; 2D VC (vitamin C-treated) + RepSox, orange; 3D control, blue; 3D VC (vitamin C-treated) + RepSox, magenta. (TIF) [file pone.0254373.s004.tif]

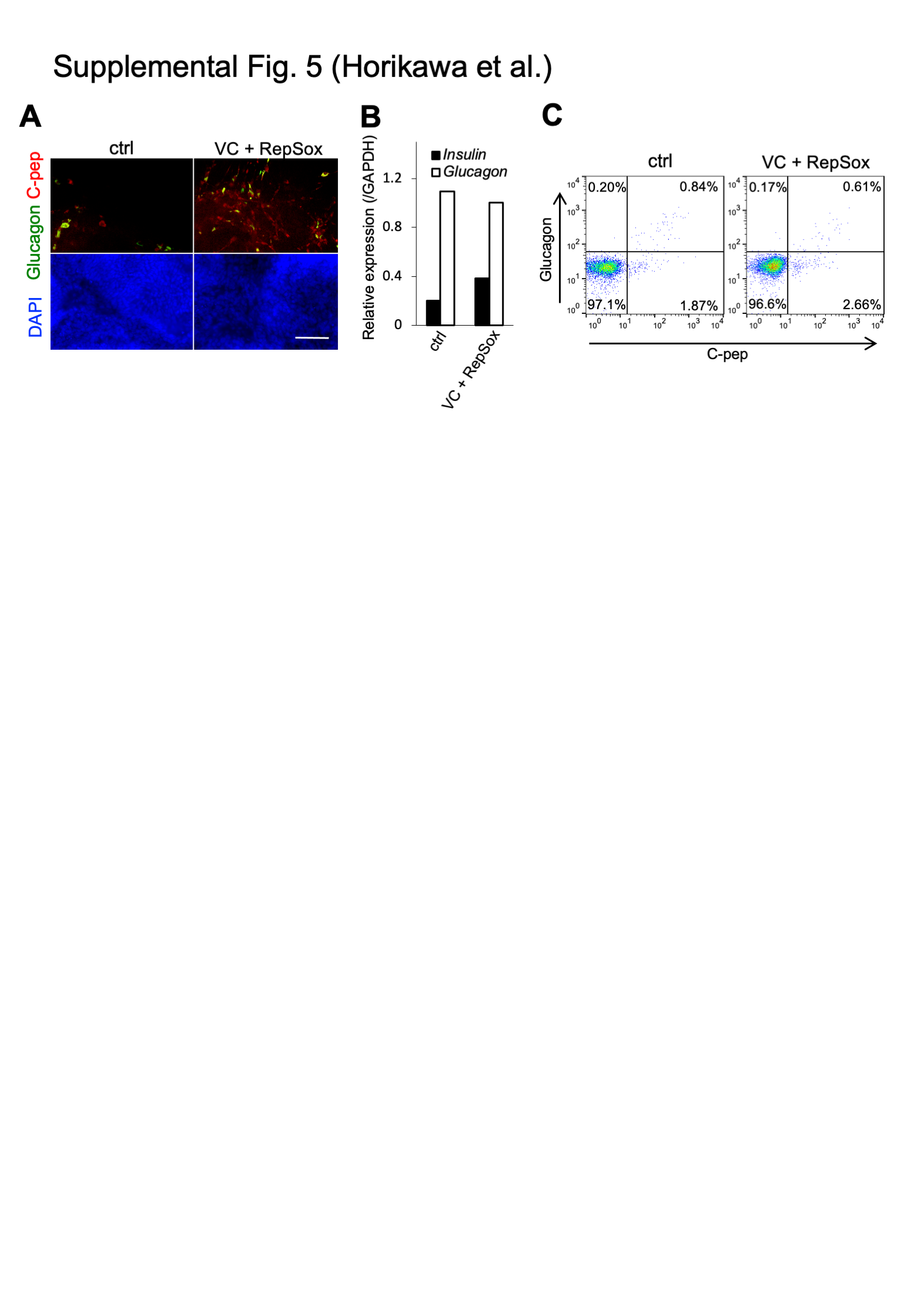

Supplement: S5 Fig — (A) Immunohistochemistry of the cells. The expression of glucagon (green) and C-peptide (red). DAPI staining shown in blue. Scale bar: 100 μm. (B) qRT-PCR analysis of the expression of Insulin (black) and Glucagon (white). (C) Flow cytometry analysis of C-peptide and glucagon expressing cells. (TIF) [file pone.0254373.s005.tif]
