# Supplementary material for: A simple method of hiPSCs differentiation into insulin-producing cells is improved with vitamin C and RepSox
Source: PLoS One. 2021 Jul 12;16(7):e0254373. doi: 10.1371/journal.pone.0254373 (PMC8274930; doi:10.1371/journal.pone.0254373)
Supplement: Attachment — Submitted filename: Horikawa_RtoR.docx [file pone.0254373.s006.docx]

**<Response to reviewers’ comments>**

Reviewer #1: First of all, I should appreciate the author’s efforts in researching in improving the protocol in the generation of iPS-derived beta cells, which I believe would be one of the sources to cure T1D in near future!

Major concerns:

The authors claimed that they provided a simple and cost-effective protocol to generate iPS-derived beta cells by emphasizing the application of Vit C and ALK-5 inhibitor and 3-D culture. Although the provided results support the idea, the application of the above-mentioned reagent and 3-D culture has been proven already by Rezania et al (Nat. biotech 2014) and Massumi et al (PLOS ONE).

The authors should compare the number of C-peptide positive cells after optimization with the above-mentioned studies to give a clear picture of their protocol vs others.

We appreciate all the reviewer’s comments. We have not directly compared the differentiation efficiency with other protocols before, so we compared with one of other methods (Yabe et al., 2017). As a result, the ratio of insulin positive cells (IHC) as well as insulin expression (qRT-PCR) in differentiated cells by our method were larger than “another” method, whereas Maf-A expression in our differentiated cells was lower than another method. Nevertheless, this result indicates that our method is basically comparable with another method. The result of the experiment was shown in S3 Fig.

Although the authors claimed that 3-D culture can increase the maturity of the cells by GSIS doesn't show such a significant difference in Fig5C. To prove the maturity the authors didn’t show the Ca2+ efflux in the differentiated cells.

Thank you for the comment. Our result indicated that insulin secretion was increased in at least 2.5 mM glucose challenge. As the reviewer points out, 25 mM challenge did not increase insulin secretion.

To evaluate the maturity of our differentiated cells, we examined Ca2+ flux in differentiated cells using Fluo-8. As a result, increase of Ca2+ flux by glucose challenge as well as KCl treatment was observed in VC + RS condition. This result is newly shown in Fig. 4H and 4I (and mentioned in main text, P13L23-30).

As it has been proven, hES is more efficient in the generation of PS-derived Beta cells, which would be more informative if they test the ES cells alongside iPS using the proposed samples.

Thank you for the comment. We understand the importance of evaluating our method using hESC. Unfortunately, we are not currently permitted to use hESC in a legal context. We would like the reviewer to understand this situation. To check cell line generality in our method, we conducted differentiation experiments using another iPS cell line (TKDN4m; S5 Fig: mentioned in main text, P16L22-24).

Lacking human islets or beta-cell lines making hard to judge the correct performance of the techniques.

As the reviewer pointing out, the comparison with intact islets or MIN6 cells is important to assess our method. We measured the quantitative level of insulin (qRT-PCR) in both human islet cells and our differentiated cells. As shown below, insulin expression level in our differentiated cells is about one-seventh. We believe that the reason for low insulin expression is due to immaturity. Also in many other methods, differentiated cells from iPSC do not necessarily show better performance in insulin expression than human islet, so we would like the reviewer to understand this common problem.

|  | relative gene expression /GAPDH |
| --- | --- |
| 2Dctrl | 0.172144 |
| 2DVCRS | 0.950453 |
| 3Dctrl | 0.261516 |
| 3DVCRS | 1.9385 |
| islet | 14.6604 |

Lack of flow cytometry to quantify the percentage of insulin-positive cells.

According to the comment, we attempted to carry out flow cytometry. Unfortunately, we could not examine using 201B7 cells (by the failure of differentiation due to a technical problem). But, we also could conduct similar experiment using TKDN4m. As a result, VC/RS addition increased the ratio of both insulin+GCN- (1.87% to 2.66%), as well as insulin+ (2.71% to 3.27%). We show the result as a supplemental figure (S5C Fig).

Minor concerns:

The quality of the pictures is low.

Sorry for inconvenience. In submission, we uploaded the figures with TIFF format. Low resolution is due to PDF conversion of the Web system. When pdf version of the manuscript is viewing, please press the button of right-upper edge of each figure.

In M&M in part of GSIS, they didn’t mention 25 mM glucose.

With reference to the comment, we revised the description of GSIS in M&M section.

The action the authors should take for re-reviewing the paper:

Confirming the percentage of the insulin-producing cells in the population by flow cytometry.

Confirming the maturity of the differentiated cells by measuring the intracellular Ca+2.

Including the ES-derived beta cells as comparing with iPS-derived beta cells

Including the human islets to confirm the accuracy of the technique and comparing with the maturity of the cells.

We thank the reviewer to summarize the point to do. We carried out (1) flowcytometry, (2) Ca2+flux assay, (3) experiment with other iPS cell lines. We also mentioned comparison with human islets. Please check each point-to-point response for detail.

Reviewer #2: The authors reported a method for differentiating insulin-producing cells from hiPSCs and improved glucose-stimulated insulin secretion by adding vitamin C and RepSox as well as by 3D culture.

The weak point of this paper is that the authors use only one cell line as hiPSCs for their experiments. It is difficult to say whether this protocol may be applicable to other hiPSCs unless they show similar data using more than two different cell lines, because hiPSCs have different characteristics.

According to the comments, we performed same experiment with another cell line (TKDN4m). The differentiation efficiency in this cell line was confirmed to be improved by vitamin C and RepSox addition (S5 Fig; mentioned in the main text, P16L22-24). In addition, we obtained insulin positive cells with same protocol using other cell line (like CiRA clinical lines), although we did not compare +/- vitamin C and RepSox.

Although the authors concluded that they could improve glucose-stimulated insulin secretion, I do not think the supporting data are enough to say so. According to the Figure 5C, C-peptide secretion decreased at 25 mM glucose concentration compared with the level at 2.5mM, which indicates the lack of glucose responsiveness. Moreover, the description of glucose-stimulated C-peptide secretion assays in M&M is not accurate.

Thanks for the reviewer’s comment. We agree that our differentiated cells showed inefficient activity to secrete insulin. To examine the maturity with another methods, we performed Ca2+ flux assay. The result showed that low and high glucose stimulation clearly increased Ca2+ level, especially in the cells differentiated with vitamin C and RepSox, suggesting that the differentiated cells at least possess the ability to respond to glucose (including high glucose challenge). Previous report showed that immature cells secrete low level of insulin continuously (Bonner-Weir and Aguayo-Mazzucato, Nature 535: 365-366 (2016)), thus we currently believe that the inefficiency of insulin secretion response to high glucose will be due to immaturity of the cells.

Also, according to the comment, we revised description of C-peptide assay in M&M section.

I agree with the authors about the necessity of cost reduction for cell therapy. Many researchers tried to induce functional islets by imitating the developmental process for pancreatic islets. It is important to show that the quality of induced βcells did not change despite the simplified method. It would be more convincing if the authors present comparison data with other reported methods to say “ This simplified method will contribute to realizing transplantation therapy of βcells using iPSCs.”

We greatly appreciate that the reviewer understands the importance of cost reduction for the regenerative therapy. We also understand that it is necessary to show the comparison with other methods. According to the comment, we carried out differentiation using one of other protocols (Yabe et al., 2017). As described above, our results indicated that the ratio of insulin positive cells as well as insulin expression in differentiated cells by our method were larger than other method, whereas Maf-A expression in our differentiated cells was lower than other method. This data is shown in S3 Fig.
